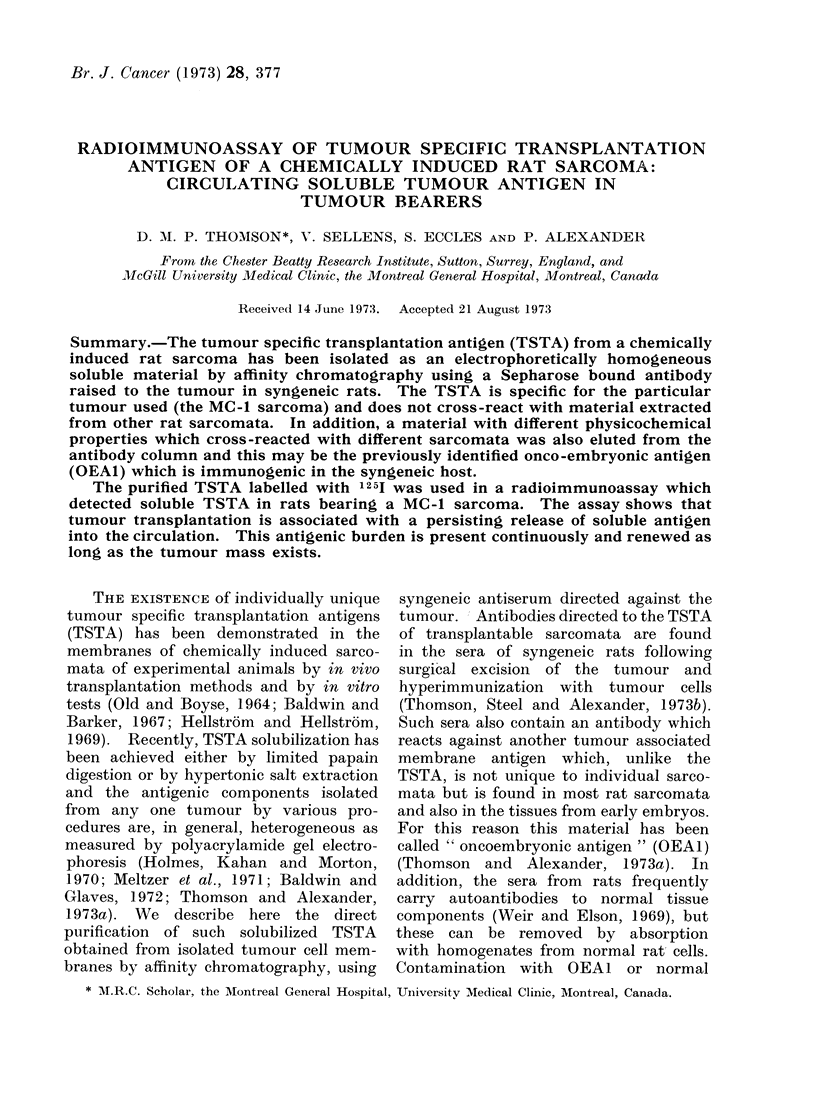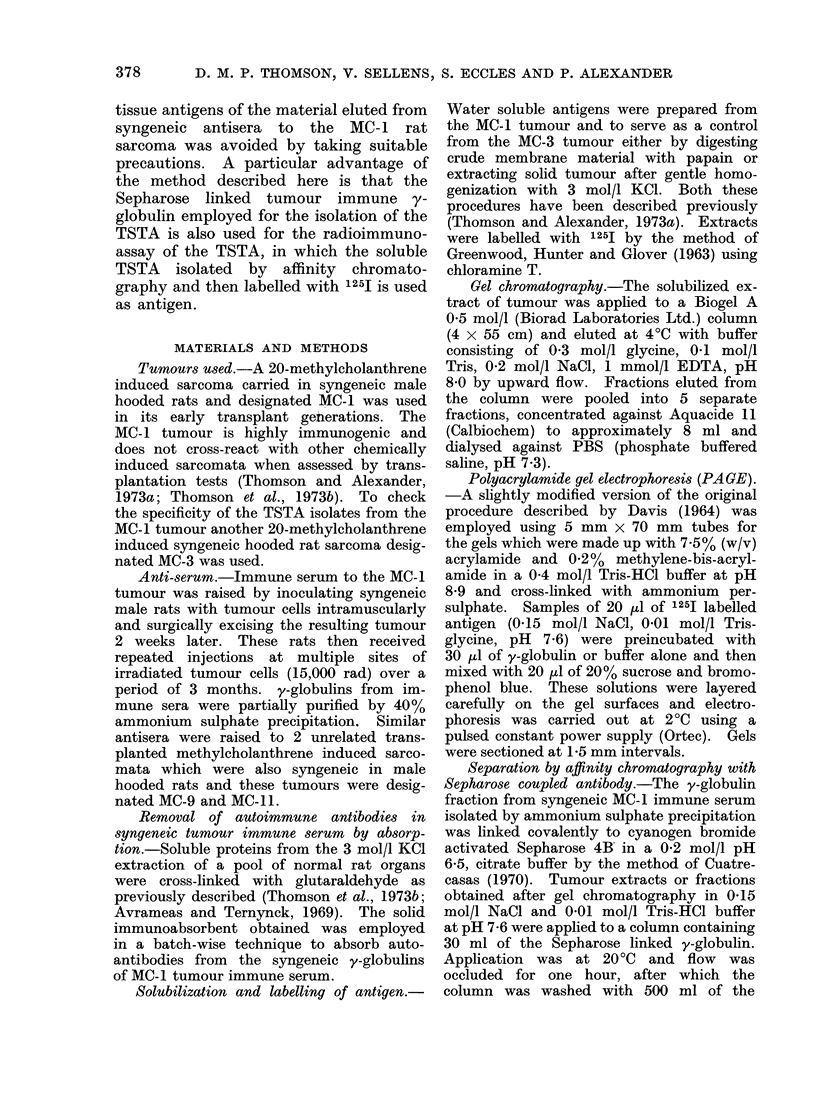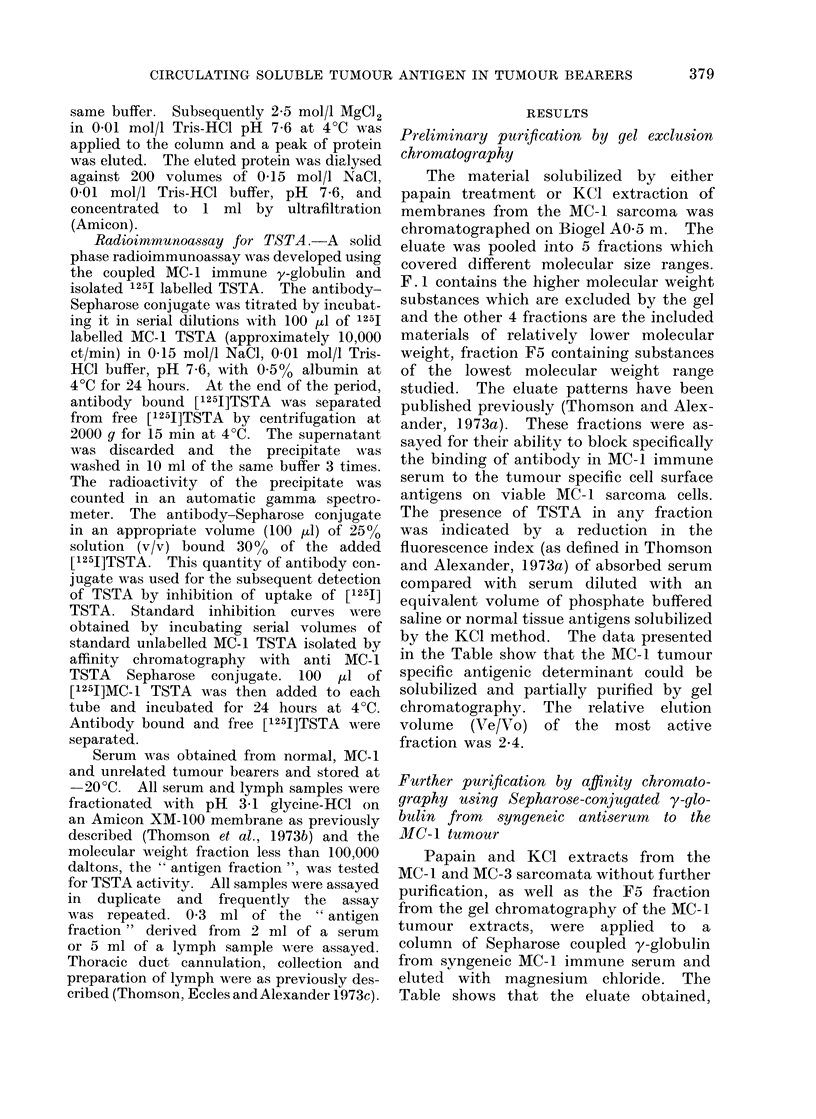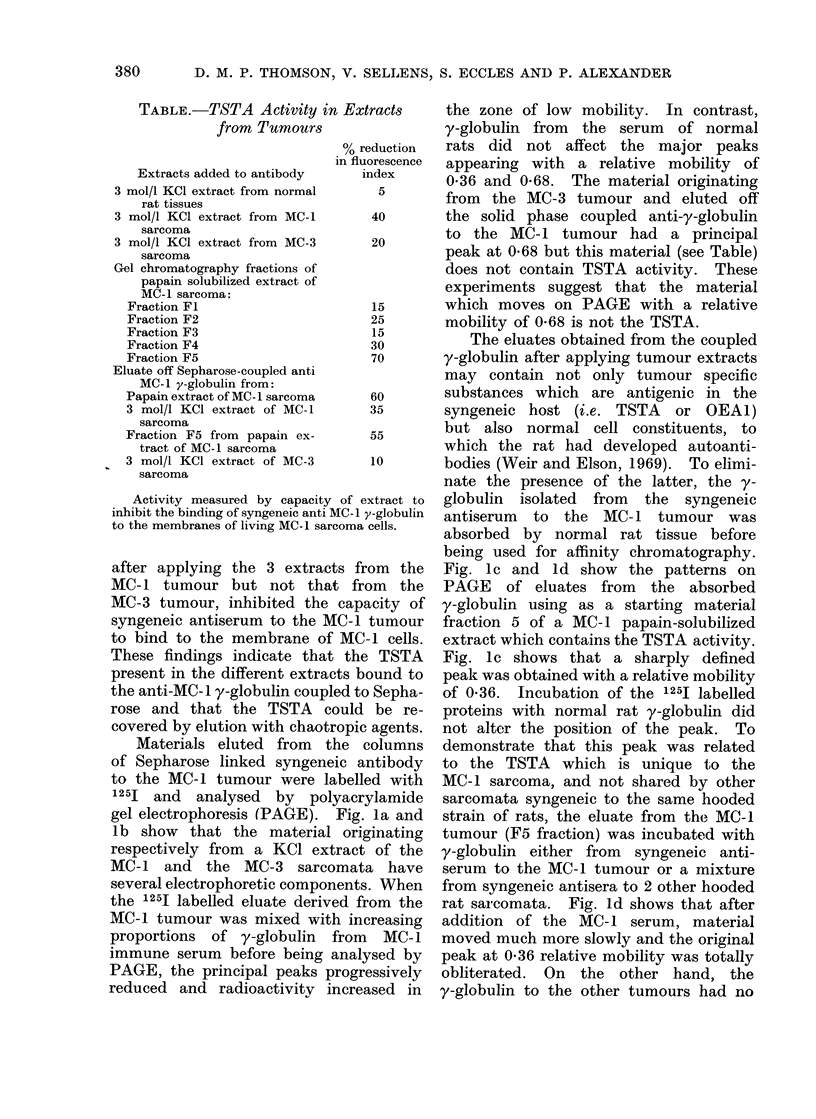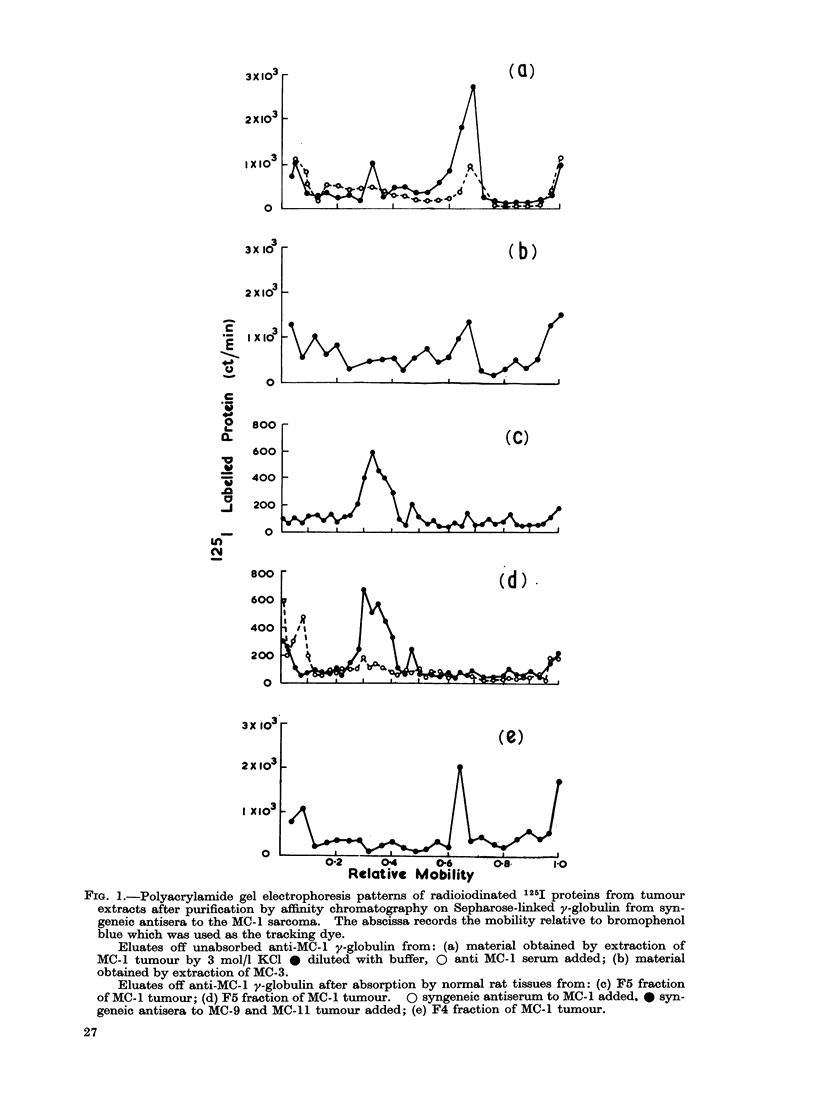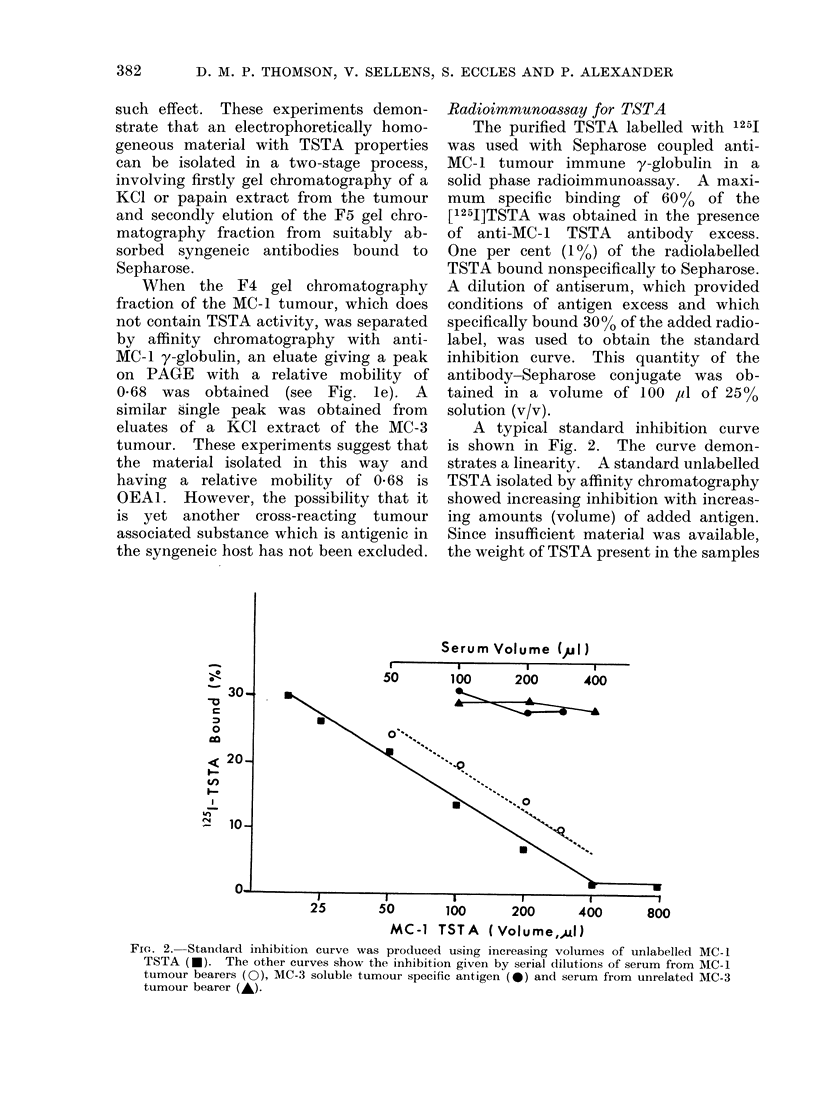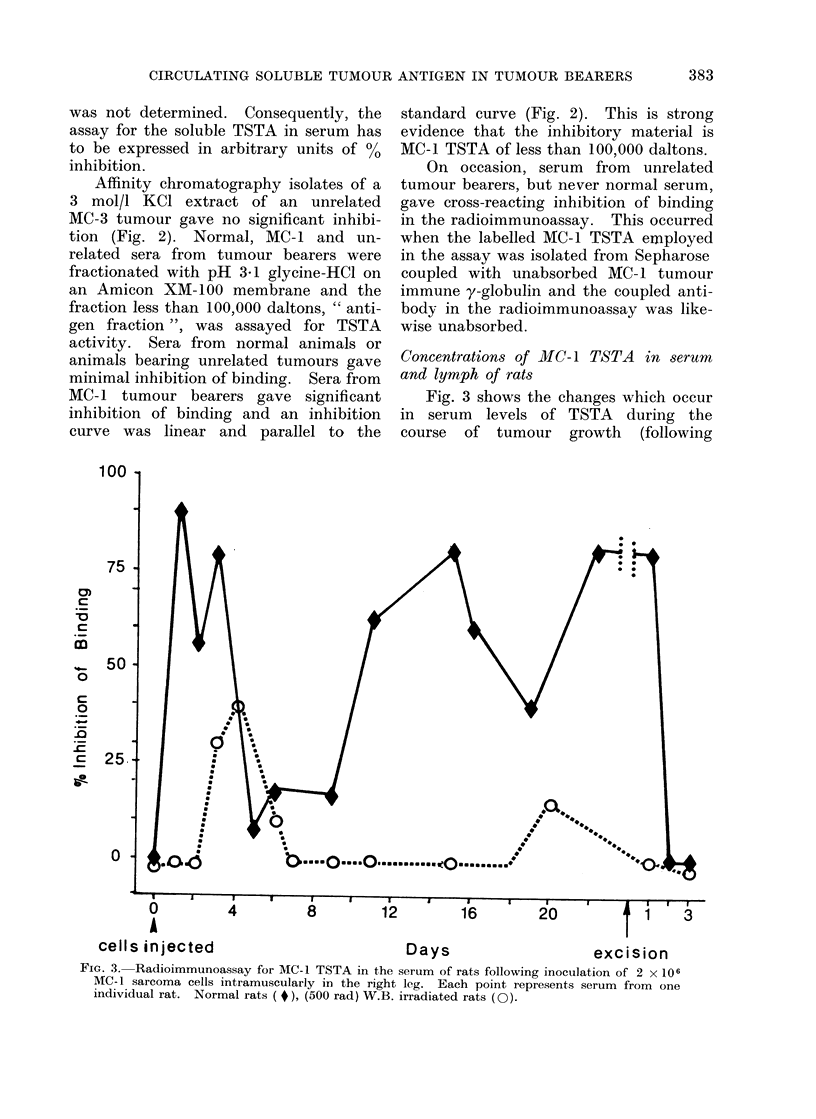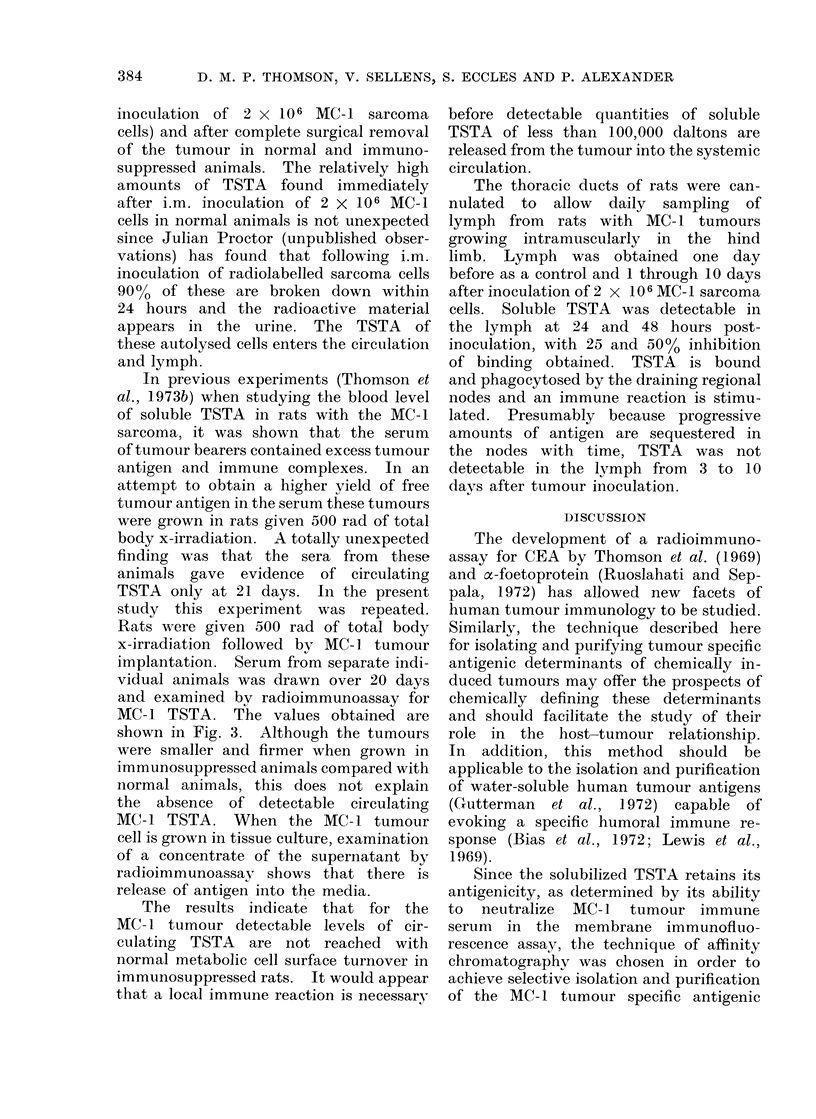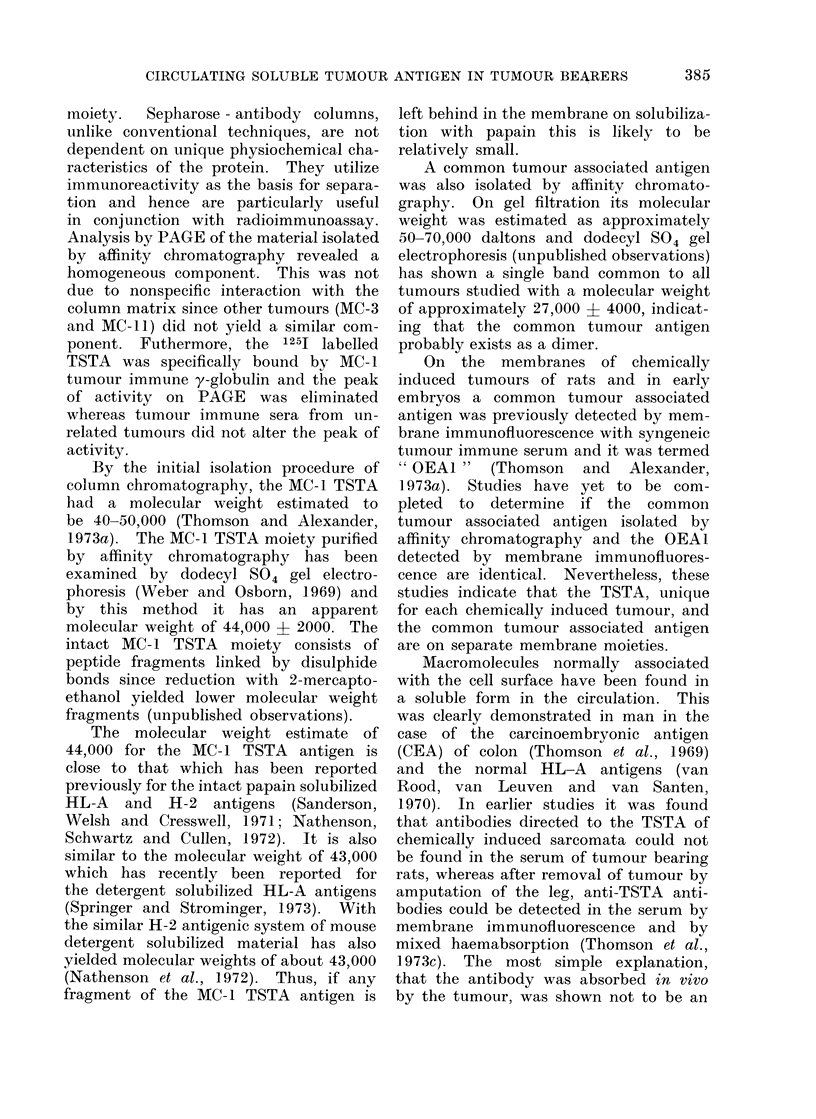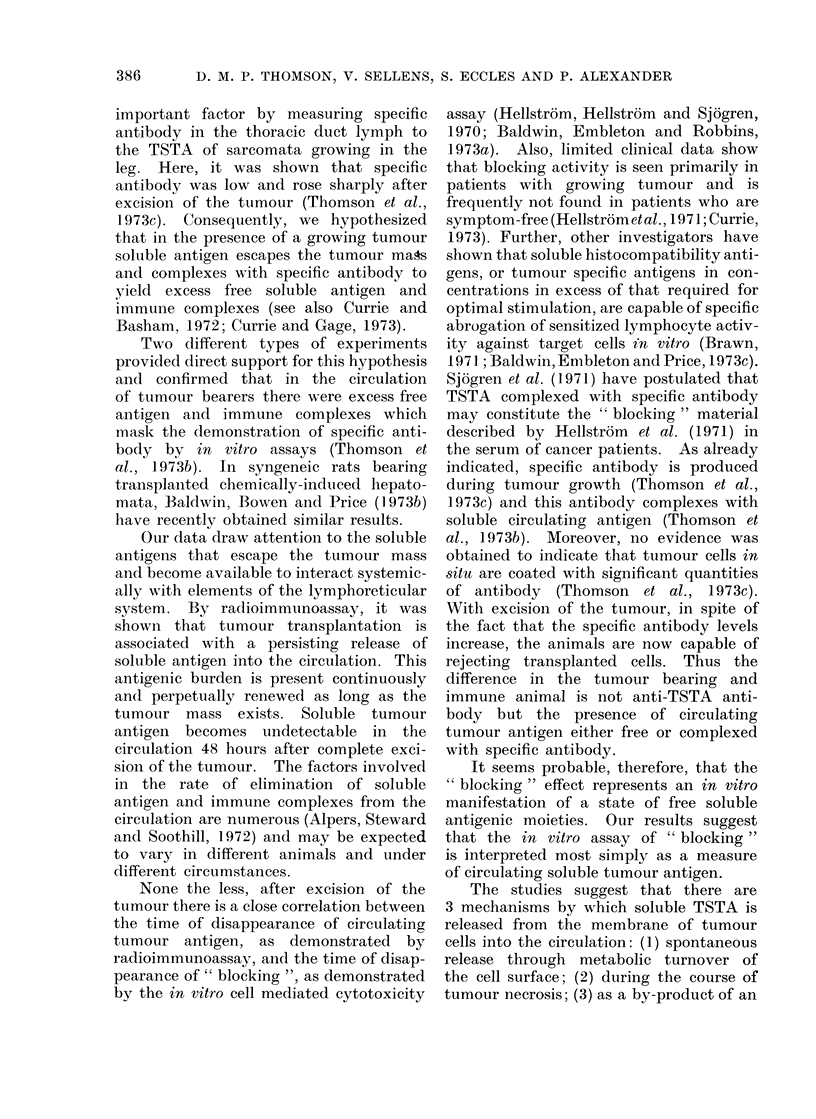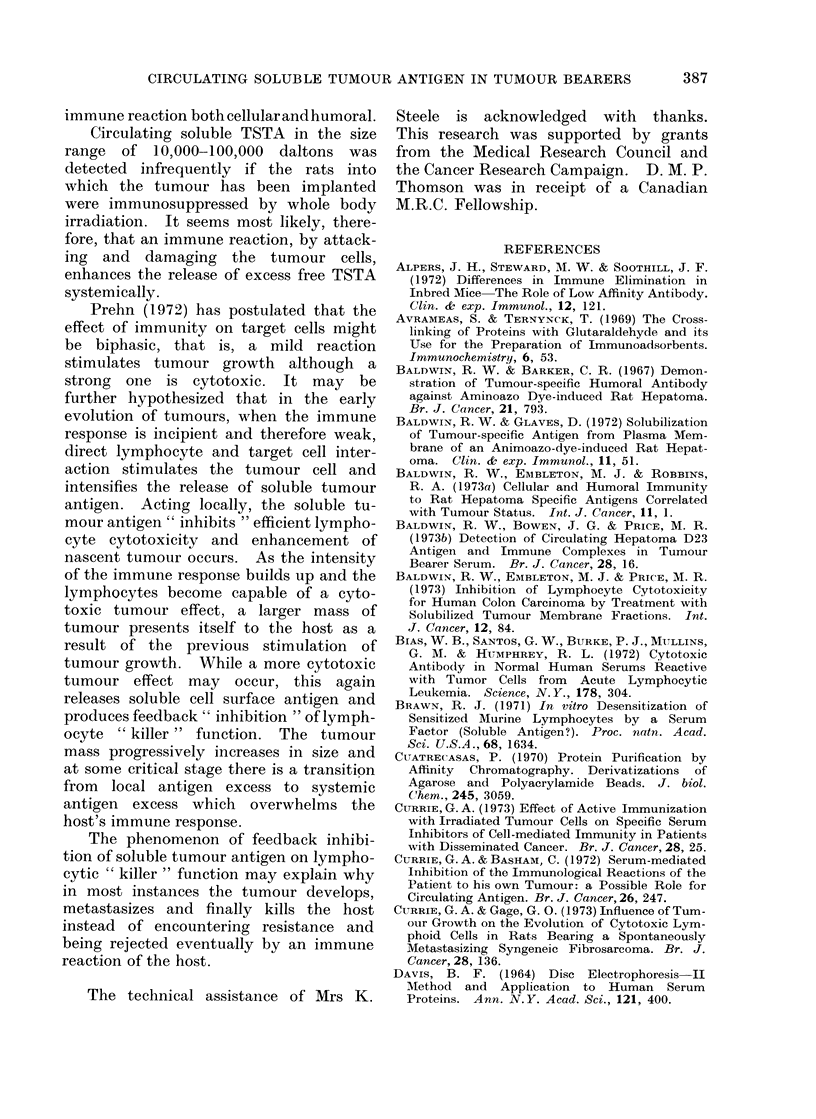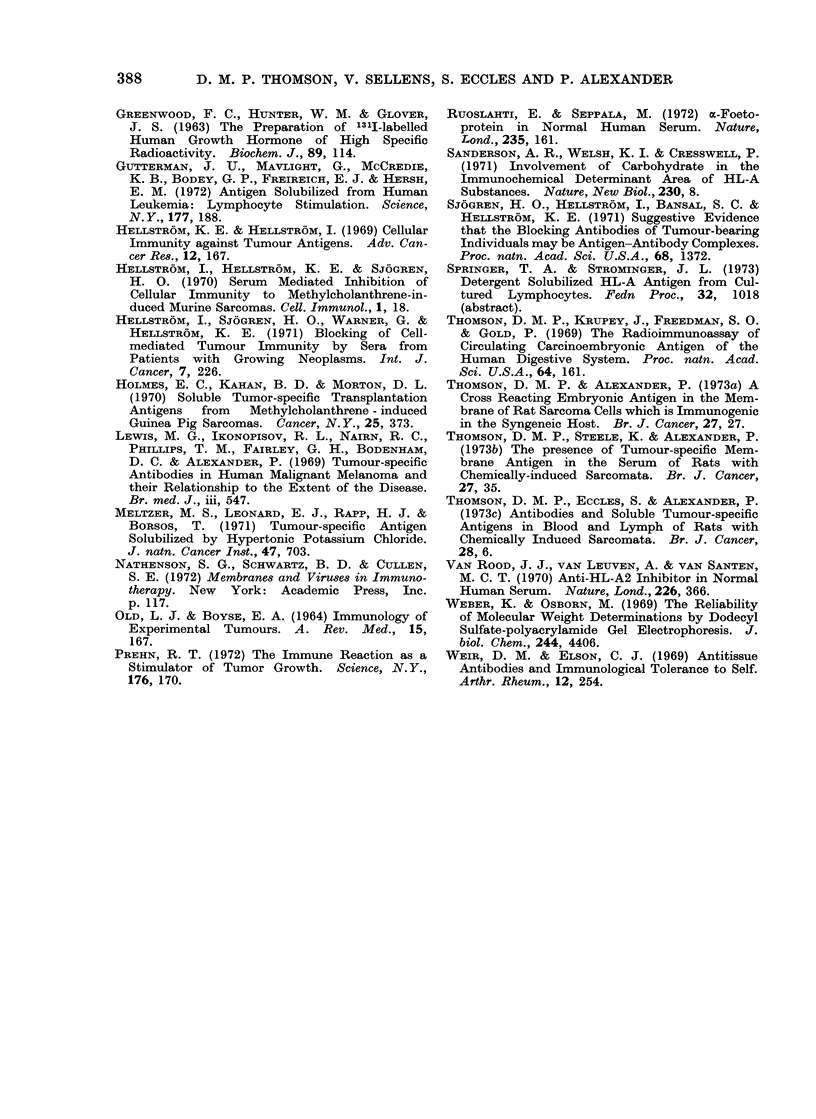# Radioimmunoassay of Tumour Specific Transplantation Antigen of a Chemically Induced Rat Sarcoma: Circulating Soluble Tumour Antigen in Tumour Bearers

**DOI:** 10.1038/bjc.1973.163

**Published:** 1973-11

**Authors:** D. M. P. Thomson, V. Sellens, S. Eccles, P. Alexander

## Abstract

The tumour specific transplantation antigen (TSTA) from a chemically induced rat sarcoma has been isolated as an electrophoretically homogeneous soluble material by affinity chromatography using a Sepharose bound antibody raised to the tumour in syngeneic rats. The TSTA is specific for the particular tumour used (the MC-1 sarcoma) and does not cross-react with material extracted from other rat sarcomata. In addition, a material with different physicochemical properties which cross-reacted with different sarcomata was also eluted from the antibody column and this may be the previously identified onco-embryonic antigen (OEA1) which is immunogenic in the syngeneic host.

The purified TSTA labelled with ^125^I was used in a radioimmunoassay which detected soluble TSTA in rats bearing a MC-1 sarcoma. The assay shows that tumour transplantation is associated with a persisting release of soluble antigen into the circulation. This antigenic burden is present continuously and renewed as long as the tumour mass exists.


*Br. J. Cancer* (1973) **28**, 377

### RADIOIMMUNOASSAY OF TUMOUR SPECIFIC TRANSPLANTATION ANTIGEN OF A CHEMICALLY INDUCED RAT SARCOMA: CIRCULATING SOLUBLE TUMOUR ANTIGEN IN TUMOUR BEARERS

D. M. P. THOMSON*, V. SELLENS, S. ECCLES AND P. ALEXANDER

*From the Chester Beatty Research Institute, Sutton, Surrey, England, and McGill University Medical Clinic, the Montreal General Hospital, Montreal, Canada*

Received 14 June 1973. Accepted 21 August 1973

**Summary.—The tumour specific transplantation antigen (TSTA) from a chemically induced rat sarcoma has been isolated as an electrophoretically homogeneous soluble material by affinity chromatography using a Sepharose bound antibody raised to the tumour in syngeneic rats. The TSTA is specific for the particular tumour used (the MC-1 sarcoma) and does not cross-react with material extracted from other rat sarcomata. In addition, a material with different physicochemical properties which cross-reacted with different sarcomata was also eluted from the antibody column and this may be the previously identified onco-embryonic antigen (OEA1) which is immunogenic in the syngeneic host.**

**The purified TSTA labelled with $^{125}I$ was used in a radioimmunoassay which detected soluble TSTA in rats bearing a MC-1 sarcoma. The assay shows that tumour transplantation is associated with a persisting release of soluble antigen into the circulation. This antigenic burden is present continuously and renewed as long as the tumour mass exists.**

THE EXISTENCE of individually unique tumour specific transplantation antigens (TSTA) has been demonstrated in the membranes of chemically induced sarcomata of experimental animals by *in vivo* transplantation methods and by *in vitro* tests (Old and Boyse, 1964; Baldwin and Barker, 1967; Hellström and Hellström, 1969). Recently, TSTA solubilization has been achieved either by limited papain digestion or by hypertonic salt extraction and the antigenic components isolated from any one tumour by various procedures are, in general, heterogeneous as measured by polyacrylamide gel electrophoresis (Holmes, Kahan and Morton, 1970; Meltzer *et al.*, 1971; Baldwin and Glaves, 1972; Thomson and Alexander, 1973*a*). We describe here the direct purification of such solubilized TSTA obtained from isolated tumour cell membranes by affinity chromatography, using syngeneic antiserum directed against the tumour. Antibodies directed to the TSTA of transplantable sarcomata are found in the sera of syngeneic rats following surgical excision of the tumour and hyperimmunization with tumour cells (Thomson, Steel and Alexander, 1973*b*). Such sera also contain an antibody which reacts against another tumour associated membrane antigen which, unlike the TSTA, is not unique to individual sarcomata but is found in most rat sarcomata and also in the tissues from early embryos. For this reason this material has been called "oncoembryonic antigen" (OEA1) (Thomson and Alexander, 1973*a*). In addition, the sera from rats frequently carry autoantibodies to normal tissue components (Weir and Elson, 1969), but these can be removed by absorption with homogenates from normal rat cells. Contamination with OEA1 or normal

* M.R.C. Scholar, the Montreal General Hospital, University Medical Clinic, Montreal, Canada.

tissue antigens of the material eluted from syngeneic antisera to the MC-1 rat sarcoma was avoided by taking suitable precautions. A particular advantage of the method described here is that the Sepharose linked tumour immune $\gamma$-globulin employed for the isolation of the TSTA is also used for the radioimmunoassay of the TSTA, in which the soluble TSTA isolated by affinity chromatography and then labelled with $^{125}I$ is used as antigen.

### MATERIALS AND METHODS

*Tumours used.*—A 20-methylcholanthrene induced sarcoma carried in syngeneic male hooded rats and designated MC-1 was used in its early transplant generations. The MC-1 tumour is highly immunogenic and does not cross-react with other chemically induced sarcomata when assessed by transplantation tests (Thomson and Alexander, 1973*a*; Thomson *et al.*, 1973*b*). To check the specificity of the TSTA isolates from the MC-1 tumour another 20-methylcholanthrene induced syngeneic hooded rat sarcoma designated MC-3 was used.

*Anti-serum.*—Immune serum to the MC-1 tumour was raised by inoculating syngeneic male rats with tumour cells intramuscularly and surgically excising the resulting tumour 2 weeks later. These rats then received repeated injections at multiple sites of irradiated tumour cells (15,000 rad) over a period of 3 months. $\gamma$-globulins from immune sera were partially purified by 40% ammonium sulphate precipitation. Similar antisera were raised to 2 unrelated transplanted methylcholanthrene induced sarcomata which were also syngeneic in male hooded rats and these tumours were designated MC-9 and MC-11.

*Removal of autoimmune antibodies in syngeneic tumour immune serum by absorption.*—Soluble proteins from the 3 mol/l KCl extraction of a pool of normal rat organs were cross-linked with glutaraldehyde as previously described (Thomson *et al.*, 1973*b*; Avrameas and Ternynck, 1969). The solid immunoabsorbent obtained was employed in a batch-wise technique to absorb autoantibodies from the syngeneic $\gamma$-globulins of MC-1 tumour immune serum.

*Solubilization and labelling of antigen.*—Water soluble antigens were prepared from the MC-1 tumour and to serve as a control from the MC-3 tumour either by digesting crude membrane material with papain or extracting solid tumour after gentle homogenization with 3 mol/l KCl. Both these procedures have been described previously (Thomson and Alexander, 1973*a*). Extracts were labelled with $^{125}I$ by the method of Greenwood, Hunter and Glover (1963) using chloramine T.

*Gel chromatography.*—The solubilized extract of tumour was applied to a Biogel A 0·5 mol/l (Biorad Laboratories Ltd.) column (4 × 55 cm) and eluted at 4°C with buffer consisting of 0·3 mol/l glycine, 0·1 mol/l Tris, 0·2 mol/l NaCl, 1 mmol/l EDTA, pH 8·0 by upward flow. Fractions eluted from the column were pooled into 5 separate fractions, concentrated against Aquacide 11 (Calbiochem) to approximately 8 ml and dialysed against PBS (phosphate buffered saline, pH 7·3).

*Polyacrylamide gel electrophoresis (PAGE).*—A slightly modified version of the original procedure described by Davis (1964) was employed using 5 mm × 70 mm tubes for the gels which were made up with 7·5% (w/v) acrylamide and 0·2% methylene-bis-acrylamide in a 0·4 mol/l Tris-HCl buffer at pH 8·9 and cross-linked with ammonium persulphate. Samples of 20 $\mu$l of $^{125}I$ labelled antigen (0·15 mol/l NaCl, 0·01 mol/l Tris-glycine, pH 7·6) were preincubated with 30 $\mu$l of $\gamma$-globulin or buffer alone and then mixed with 20 $\mu$l of 20% sucrose and bromophenol blue. These solutions were layered carefully on the gel surfaces and electrophoresis was carried out at 2°C using a pulsed constant power supply (Ortec). Gels were sectioned at 1·5 mm intervals.

*Separation by affinity chromatography with Sepharose coupled antibody.*—The $\gamma$-globulin fraction from syngeneic MC-1 immune serum isolated by ammonium sulphate precipitation was linked covalently to cyanogen bromide activated Sepharose 4B in a 0·2 mol/l pH 6·5, citrate buffer by the method of Cuatrecasas (1970). Tumour extracts or fractions obtained after gel chromatography in 0·15 mol/l NaCl and 0·01 mol/l Tris-HCl buffer at pH 7·6 were applied to a column containing 30 ml of the Sepharose linked $\gamma$-globulin. Application was at 20°C and flow was occluded for one hour, after which the column was washed with 500 ml of the same buffer. Subsequently 2·5 mol/l $MgCl_2$ in 0·01 mol/l Tris-HCl pH 7·6 at 4°C was applied to the column and a peak of protein was eluted. The eluted protein was dialysed against 200 volumes of 0·15 mol/l NaCl, 0·01 mol/l Tris-HCl buffer, pH 7·6, and concentrated to 1 ml by ultrafiltration (Amicon).

*Radioimmunoassay for TSTA.*—A solid phase radioimmunoassay was developed using the coupled MC-1 immune γ-globulin and isolated $^{125}I$ labelled TSTA. The antibody–Sepharose conjugate was titrated by incubating it in serial dilutions with 100 μl of $^{125}I$ labelled MC-1 TSTA (approximately 10,000 ct/min) in 0·15 mol/l NaCl, 0·01 mol/l Tris-HCl buffer, pH 7·6, with 0·5% albumin at 4°C for 24 hours. At the end of the period, antibody bound [$^{125}I$]TSTA was separated from free [$^{125}I$]TSTA by centrifugation at 2000 *g* for 15 min at 4°C. The supernatant was discarded and the precipitate was washed in 10 ml of the same buffer 3 times. The radioactivity of the precipitate was counted in an automatic gamma spectrometer. The antibody–Sepharose conjugate in an appropriate volume (100 μl) of 25% solution (v/v) bound 30% of the added [$^{125}I$]TSTA. This quantity of antibody conjugate was used for the subsequent detection of TSTA by inhibition of uptake of [$^{125}I$] TSTA. Standard inhibition curves were obtained by incubating serial volumes of standard unlabelled MC-1 TSTA isolated by affinity chromatography with anti MC-1 TSTA Sepharose conjugate. 100 μl of [$^{125}I$]MC-1 TSTA was then added to each tube and incubated for 24 hours at 4°C. Antibody bound and free [$^{125}I$]TSTA were separated.

Serum was obtained from normal, MC-1 and unrelated tumour bearers and stored at −20°C. All serum and lymph samples were fractionated with pH 3·1 glycine-HCl on an Amicon XM-100 membrane as previously described (Thomson *et al.*, 1973*b*) and the molecular weight fraction less than 100,000 daltons, the "antigen fraction", was tested for TSTA activity. All samples were assayed in duplicate and frequently the assay was repeated. 0·3 ml of the "antigen fraction" derived from 2 ml of a serum or 5 ml of a lymph sample were assayed. Thoracic duct cannulation, collection and preparation of lymph were as previously described (Thomson, Eccles and Alexander 1973*c*).

### RESULTS

### *Preliminary purification by gel exclusion chromatography*

The material solubilized by either papain treatment or KCl extraction of membranes from the MC-1 sarcoma was chromatographed on Biogel A0·5 m. The eluate was pooled into 5 fractions which covered different molecular size ranges. F. 1 contains the higher molecular weight substances which are excluded by the gel and the other 4 fractions are the included materials of relatively lower molecular weight, fraction F5 containing substances of the lowest molecular weight range studied. The eluate patterns have been published previously (Thomson and Alexander, 1973*a*). These fractions were assayed for their ability to block specifically the binding of antibody in MC-1 immune serum to the tumour specific cell surface antigens on viable MC-1 sarcoma cells. The presence of TSTA in any fraction was indicated by a reduction in the fluorescence index (as defined in Thomson and Alexander, 1973*a*) of absorbed serum compared with serum diluted with an equivalent volume of phosphate buffered saline or normal tissue antigens solubilized by the KCl method. The data presented in the Table show that the MC-1 tumour specific antigenic determinant could be solubilized and partially purified by gel chromatography. The relative elution volume (Ve/Vo) of the most active fraction was 2·4.

### *Further purification by affinity chromatography using Sepharose-conjugated γ-globulin from syngeneic antiserum to the MC-1 tumour*

Papain and KCl extracts from the MC-1 and MC-3 sarcomata without further purification, as well as the F5 fraction from the gel chromatography of the MC-1 tumour extracts, were applied to a column of Sepharose coupled γ-globulin from syngeneic MC-1 immune serum and eluted with magnesium chloride. The Table shows that the eluate obtained,

TABLE.—*TSTA Activity in Extracts from Tumours*

| Extracts added to antibody | % reduction in fluorescence index |
|---|---|
| 3 mol/l KCl extract from normal rat tissues | 5 |
| 3 mol/l KCl extract from MC-1 sarcoma | 40 |
| 3 mol/l KCl extract from MC-3 sarcoma | 20 |
| Gel chromatography fractions of papain solubilized extract of MC-1 sarcoma: | |
| Fraction F1 | 15 |
| Fraction F2 | 25 |
| Fraction F3 | 15 |
| Fraction F4 | 30 |
| Fraction F5 | 70 |
| Eluate off Sepharose-coupled anti MC-1 $\gamma$-globulin from: | |
| Papain extract of MC-1 sarcoma | 60 |
| 3 mol/l KCl extract of MC-1 sarcoma | 35 |
| Fraction F5 from papain extract of MC-1 sarcoma | 55 |
| 3 mol/l KCl extract of MC-3 sarcoma | 10 |

Activity measured by capacity of extract to inhibit the binding of syngeneic anti MC-1 $\gamma$-globulin to the membranes of living MC-1 sarcoma cells.

after applying the 3 extracts from the MC-1 tumour but not that from the MC-3 tumour, inhibited the capacity of syngeneic antiserum to the MC-1 tumour to bind to the membrane of MC-1 cells. These findings indicate that the TSTA present in the different extracts bound to the anti-MC-1 $\gamma$-globulin coupled to Sepharose and that the TSTA could be recovered by elution with chaotropic agents.

Materials eluted from the columns of Sepharose linked syngeneic antibody to the MC-1 tumour were labelled with $^{125}I$ and analysed by polyacrylamide gel electrophoresis (PAGE). Fig. 1a and 1b show that the material originating respectively from a KCl extract of the MC-1 and the MC-3 sarcomata have several electrophoretic components. When the $^{125}I$ labelled eluate derived from the MC-1 tumour was mixed with increasing proportions of $\gamma$-globulin from MC-1 immune serum before being analysed by PAGE, the principal peaks progressively reduced and radioactivity increased in the zone of low mobility. In contrast, $\gamma$-globulin from the serum of normal rats did not affect the major peaks appearing with a relative mobility of 0·36 and 0·68. The material originating from the MC-3 tumour and eluted off the solid phase coupled anti-$\gamma$-globulin to the MC-1 tumour had a principal peak at 0·68 but this material (see Table) does not contain TSTA activity. These experiments suggest that the material which moves on PAGE with a relative mobility of 0·68 is not the TSTA.

The eluates obtained from the coupled $\gamma$-globulin after applying tumour extracts may contain not only tumour specific substances which are antigenic in the syngeneic host (*i.e.* TSTA or OEA1) but also normal cell constituents, to which the rat had developed autoantibodies (Weir and Elson, 1969). To eliminate the presence of the latter, the $\gamma$-globulin isolated from the syngeneic antiserum to the MC-1 tumour was absorbed by normal rat tissue before being used for affinity chromatography. Fig. 1c and 1d show the patterns on PAGE of eluates from the absorbed $\gamma$-globulin using as a starting material fraction 5 of a MC-1 papain-solubilized extract which contains the TSTA activity. Fig. 1c shows that a sharply defined peak was obtained with a relative mobility of 0·36. Incubation of the $^{125}I$ labelled proteins with normal rat $\gamma$-globulin did not alter the position of the peak. To demonstrate that this peak was related to the TSTA which is unique to the MC-1 sarcoma, and not shared by other sarcomata syngeneic to the same hooded strain of rats, the eluate from the MC-1 tumour (F5 fraction) was incubated with $\gamma$-globulin either from syngeneic antiserum to the MC-1 tumour or a mixture from syngeneic antisera to 2 other hooded rat sarcomata. Fig. 1d shows that after addition of the MC-1 serum, material moved much more slowly and the original peak at 0·36 relative mobility was totally obliterated. On the other hand, the $\gamma$-globulin to the other tumours had no

FIG. 1.—Polyacrylamide gel electrophoresis patterns of radioiodinated $^{125}$I proteins from tumour extracts after purification by affinity chromatography on Sepharose-linked $\gamma$-globulin from syngeneic antisera to the MC-1 sarcoma. The abscissa records the mobility relative to bromophenol blue which was used as the tracking dye.

Eluates off unabsorbed anti-MC-1 $\gamma$-globulin from: (a) material obtained by extraction of MC-1 tumour by 3 mol/l KCl ● diluted with buffer, ○ anti MC-1 serum added; (b) material obtained by extraction of MC-3.

Eluates off anti-MC-1 $\gamma$-globulin after absorption by normal rat tissues from: (c) F5 fraction of MC-1 tumour; (d) F5 fraction of MC-1 tumour. ○ syngeneic antiserum to MC-1 added, ● syngeneic antisera to MC-9 and MC-11 tumour added; (e) F4 fraction of MC-1 tumour.

such effect. These experiments demonstrate that an electrophoretically homogeneous material with TSTA properties can be isolated in a two-stage process, involving firstly gel chromatography of a KCl or papain extract from the tumour and secondly elution of the F5 gel chromatography fraction from suitably absorbed syngeneic antibodies bound to Sepharose.

When the F4 gel chromatography fraction of the MC-1 tumour, which does not contain TSTA activity, was separated by affinity chromatography with anti-MC-1 $\gamma$-globulin, an eluate giving a peak on PAGE with a relative mobility of 0·68 was obtained (see Fig. 1e). A similar single peak was obtained from eluates of a KCl extract of the MC-3 tumour. These experiments suggest that the material isolated in this way and having a relative mobility of 0·68 is OEA1. However, the possibility that it is yet another cross-reacting tumour associated substance which is antigenic in the syngeneic host has not been excluded.

*Radioimmunoassay for TSTA*

The purified TSTA labelled with $^{125}I$ was used with Sepharose coupled anti-MC-1 tumour immune $\gamma$-globulin in a solid phase radioimmunoassay. A maximum specific binding of 60% of the [$^{125}I$]TSTA was obtained in the presence of anti-MC-1 TSTA antibody excess. One per cent (1%) of the radiolabelled TSTA bound nonspecifically to Sepharose. A dilution of antiserum, which provided conditions of antigen excess and which specifically bound 30% of the added radiolabel, was used to obtain the standard inhibition curve. This quantity of the antibody–Sepharose conjugate was obtained in a volume of 100 $\mu$l of 25% solution (v/v).

A typical standard inhibition curve is shown in Fig. 2. The curve demonstrates a linearity. A standard unlabelled TSTA isolated by affinity chromatography showed increasing inhibition with increasing amounts (volume) of added antigen. Since insufficient material was available, the weight of TSTA present in the samples

FIG. 2.—Standard inhibition curve was produced using increasing volumes of unlabelled MC-1 TSTA (■). The other curves show the inhibition given by serial dilutions of serum from MC-1 tumour bearers (○), MC-3 soluble tumour specific antigen (●) and serum from unrelated MC-3 tumour bearer (▲).

was not determined. Consequently, the assay for the soluble TSTA in serum has to be expressed in arbitrary units of % inhibition.

Affinity chromatography isolates of a 3 mol/l KCl extract of an unrelated MC-3 tumour gave no significant inhibition (Fig. 2). Normal, MC-1 and unrelated sera from tumour bearers were fractionated with pH 3·1 glycine-HCl on an Amicon XM-100 membrane and the fraction less than 100,000 daltons, " antigen fraction ", was assayed for TSTA activity. Sera from normal animals or animals bearing unrelated tumours gave minimal inhibition of binding. Sera from MC-1 tumour bearers gave significant inhibition of binding and an inhibition curve was linear and parallel to the standard curve (Fig. 2). This is strong evidence that the inhibitory material is MC-1 TSTA of less than 100,000 daltons.

On occasion, serum from unrelated tumour bearers, but never normal serum, gave cross-reacting inhibition of binding in the radioimmunoassay. This occurred when the labelled MC-1 TSTA employed in the assay was isolated from Sepharose coupled with unabsorbed MC-1 tumour immune $\gamma$-globulin and the coupled antibody in the radioimmunoassay was likewise unabsorbed.

*Concentrations of MC-1 TSTA in serum and lymph of rats*

Fig. 3 shows the changes which occur in serum levels of TSTA during the course of tumour growth (following

FIG. 3.—Radioimmunoassay for MC-1 TSTA in the serum of rats following inoculation of $2 \times 10^6$ MC-1 sarcoma cells intramuscularly in the right leg. Each point represents serum from one individual rat. Normal rats (♦), (500 rad) W.B. irradiated rats (○).

inoculation of $2 \times 10^6$ MC-1 sarcoma cells) and after complete surgical removal of the tumour in normal and immunosuppressed animals. The relatively high amounts of TSTA found immediately after i.m. inoculation of $2 \times 10^6$ MC-1 cells in normal animals is not unexpected since Julian Proctor (unpublished observations) has found that following i.m. inoculation of radiolabelled sarcoma cells 90% of these are broken down within 24 hours and the radioactive material appears in the urine. The TSTA of these autolysed cells enters the circulation and lymph.

In previous experiments (Thomson *et al.*, 1973*b*) when studying the blood level of soluble TSTA in rats with the MC-1 sarcoma, it was shown that the serum of tumour bearers contained excess tumour antigen and immune complexes. In an attempt to obtain a higher yield of free tumour antigen in the serum these tumours were grown in rats given 500 rad of total body x-irradiation. A totally unexpected finding was that the sera from these animals gave evidence of circulating TSTA only at 21 days. In the present study this experiment was repeated. Rats were given 500 rad of total body x-irradiation followed by MC-1 tumour implantation. Serum from separate individual animals was drawn over 20 days and examined by radioimmunoassay for MC-1 TSTA. The values obtained are shown in Fig. 3. Although the tumours were smaller and firmer when grown in immunosuppressed animals compared with normal animals, this does not explain the absence of detectable circulating MC-1 TSTA. When the MC-1 tumour cell is grown in tissue culture, examination of a concentrate of the supernatant by radioimmunoassay shows that there is release of antigen into the media.

The results indicate that for the MC-1 tumour detectable levels of circulating TSTA are not reached with normal metabolic cell surface turnover in immunosuppressed rats. It would appear that a local immune reaction is necessary before detectable quantities of soluble TSTA of less than 100,000 daltons are released from the tumour into the systemic circulation.

The thoracic ducts of rats were cannulated to allow daily sampling of lymph from rats with MC-1 tumours growing intramuscularly in the hind limb. Lymph was obtained one day before as a control and 1 through 10 days after inoculation of $2 \times 10^6$ MC-1 sarcoma cells. Soluble TSTA was detectable in the lymph at 24 and 48 hours post-inoculation, with 25 and 50% inhibition of binding obtained. TSTA is bound and phagocytosed by the draining regional nodes and an immune reaction is stimulated. Presumably because progressive amounts of antigen are sequestered in the nodes with time, TSTA was not detectable in the lymph from 3 to 10 days after tumour inoculation.

### DISCUSSION

The development of a radioimmunoassay for CEA by Thomson *et al.* (1969) and $\alpha$-foetoprotein (Ruoslahati and Seppala, 1972) has allowed new facets of human tumour immunology to be studied. Similarly, the technique described here for isolating and purifying tumour specific antigenic determinants of chemically induced tumours may offer the prospects of chemically defining these determinants and should facilitate the study of their role in the host–tumour relationship. In addition, this method should be applicable to the isolation and purification of water-soluble human tumour antigens (Gutterman *et al.*, 1972) capable of evoking a specific humoral immune response (Bias *et al.*, 1972; Lewis *et al.*, 1969).

Since the solubilized TSTA retains its antigenicity, as determined by its ability to neutralize MC-1 tumour immune serum in the membrane immunofluorescence assay, the technique of affinity chromatography was chosen in order to achieve selective isolation and purification of the MC-1 tumour specific antigenic moiety. Sepharose - antibody columns, unlike conventional techniques, are not dependent on unique physiochemical characteristics of the protein. They utilize immunoreactivity as the basis for separation and hence are particularly useful in conjunction with radioimmunoassay. Analysis by PAGE of the material isolated by affinity chromatography revealed a homogeneous component. This was not due to nonspecific interaction with the column matrix since other tumours (MC-3 and MC-11) did not yield a similar component. Futhermore, the $^{125}I$ labelled TSTA was specifically bound by MC-1 tumour immune $\gamma$-globulin and the peak of activity on PAGE was eliminated whereas tumour immune sera from unrelated tumours did not alter the peak of activity.

By the initial isolation procedure of column chromatography, the MC-1 TSTA had a molecular weight estimated to be 40–50,000 (Thomson and Alexander, 1973*a*). The MC-1 TSTA moiety purified by affinity chromatography has been examined by dodecyl $SO_4$ gel electrophoresis (Weber and Osborn, 1969) and by this method it has an apparent molecular weight of 44,000 $\pm$ 2000. The intact MC-1 TSTA moiety consists of peptide fragments linked by disulphide bonds since reduction with 2-mercaptoethanol yielded lower molecular weight fragments (unpublished observations).

The molecular weight estimate of 44,000 for the MC-1 TSTA antigen is close to that which has been reported previously for the intact papain solubilized HL-A and H-2 antigens (Sanderson, Welsh and Cresswell, 1971; Nathenson, Schwartz and Cullen, 1972). It is also similar to the molecular weight of 43,000 which has recently been reported for the detergent solubilized HL-A antigens (Springer and Strominger, 1973). With the similar H-2 antigenic system of mouse detergent solubilized material has also yielded molecular weights of about 43,000 (Nathenson *et al.*, 1972). Thus, if any fragment of the MC-1 TSTA antigen is left behind in the membrane on solubilization with papain this is likely to be relatively small.

A common tumour associated antigen was also isolated by affinity chromatography. On gel filtration its molecular weight was estimated as approximately 50–70,000 daltons and dodecyl $SO_4$ gel electrophoresis (unpublished observations) has shown a single band common to all tumours studied with a molecular weight of approximately 27,000 $\pm$ 4000, indicating that the common tumour antigen probably exists as a dimer.

On the membranes of chemically induced tumours of rats and in early embryos a common tumour associated antigen was previously detected by membrane immunofluorescence with syngeneic tumour immune serum and it was termed "OEA1" (Thomson and Alexander, 1973*a*). Studies have yet to be completed to determine if the common tumour associated antigen isolated by affinity chromatography and the OEA1 detected by membrane immunofluorescence are identical. Nevertheless, these studies indicate that the TSTA, unique for each chemically induced tumour, and the common tumour associated antigen are on separate membrane moieties.

Macromolecules normally associated with the cell surface have been found in a soluble form in the circulation. This was clearly demonstrated in man in the case of the carcinoembryonic antigen (CEA) of colon (Thomson *et al.*, 1969) and the normal HL–A antigens (van Rood, van Leuven and van Santen, 1970). In earlier studies it was found that antibodies directed to the TSTA of chemically induced sarcomata could not be found in the serum of tumour bearing rats, whereas after removal of tumour by amputation of the leg, anti-TSTA antibodies could be detected in the serum by membrane immunofluorescence and by mixed haemabsorption (Thomson *et al.*, 1973*c*). The most simple explanation, that the antibody was absorbed *in vivo* by the tumour, was shown not to be an important factor by measuring specific antibody in the thoracic duct lymph to the TSTA of sarcomata growing in the leg. Here, it was shown that specific antibody was low and rose sharply after excision of the tumour (Thomson *et al.*, 1973*c*). Consequently, we hypothesized that in the presence of a growing tumour soluble antigen escapes the tumour mass and complexes with specific antibody to yield excess free soluble antigen and immune complexes (see also Currie and Basham, 1972; Currie and Gage, 1973).

Two different types of experiments provided direct support for this hypothesis and confirmed that in the circulation of tumour bearers there were excess free antigen and immune complexes which mask the demonstration of specific antibody by *in vitro* assays (Thomson *et al.*, 1973*b*). In syngeneic rats bearing transplanted chemically-induced hepatomata, Baldwin, Bowen and Price (1973*b*) have recently obtained similar results.

Our data draw attention to the soluble antigens that escape the tumour mass and become available to interact systemically with elements of the lymphoreticular system. By radioimmunoassay, it was shown that tumour transplantation is associated with a persisting release of soluble antigen into the circulation. This antigenic burden is present continuously and perpetually renewed as long as the tumour mass exists. Soluble tumour antigen becomes undetectable in the circulation 48 hours after complete excision of the tumour. The factors involved in the rate of elimination of soluble antigen and immune complexes from the circulation are numerous (Alpers, Steward and Soothill, 1972) and may be expected to vary in different animals and under different circumstances.

None the less, after excision of the tumour there is a close correlation between the time of disappearance of circulating tumour antigen, as demonstrated by radioimmunoassay, and the time of disappearance of " blocking ", as demonstrated by the *in vitro* cell mediated cytotoxicity assay (Hellström, Hellström and Sjögren, 1970; Baldwin, Embleton and Robbins, 1973*a*). Also, limited clinical data show that blocking activity is seen primarily in patients with growing tumour and is frequently not found in patients who are symptom-free (Hellström *et al.*, 1971; Currie, 1973). Further, other investigators have shown that soluble histocompatibility antigens, or tumour specific antigens in concentrations in excess of that required for optimal stimulation, are capable of specific abrogation of sensitized lymphocyte activity against target cells *in vitro* (Brawn, 1971; Baldwin, Embleton and Price, 1973*c*). Sjögren *et al.* (1971) have postulated that TSTA complexed with specific antibody may constitute the " blocking " material described by Hellström *et al.* (1971) in the serum of cancer patients. As already indicated, specific antibody is produced during tumour growth (Thomson *et al.*, 1973*c*) and this antibody complexes with soluble circulating antigen (Thomson *et al.*, 1973*b*). Moreover, no evidence was obtained to indicate that tumour cells *in situ* are coated with significant quantities of antibody (Thomson *et al.*, 1973*c*). With excision of the tumour, in spite of the fact that the specific antibody levels increase, the animals are now capable of rejecting transplanted cells. Thus the difference in the tumour bearing and immune animal is not anti-TSTA antibody but the presence of circulating tumour antigen either free or complexed with specific antibody.

It seems probable, therefore, that the " blocking " effect represents an *in vitro* manifestation of a state of free soluble antigenic moieties. Our results suggest that the *in vitro* assay of " blocking " is interpreted most simply as a measure of circulating soluble tumour antigen.

The studies suggest that there are 3 mechanisms by which soluble TSTA is released from the membrane of tumour cells into the circulation: (1) spontaneous release through metabolic turnover of the cell surface; (2) during the course of tumour necrosis; (3) as a by-product of an immune reaction both cellular and humoral.

Circulating soluble TSTA in the size range of 10,000–100,000 daltons was detected infrequently if the rats into which the tumour has been implanted were immunosuppressed by whole body irradiation. It seems most likely, therefore, that an immune reaction, by attacking and damaging the tumour cells, enhances the release of excess free TSTA systemically.

Prehn (1972) has postulated that the effect of immunity on target cells might be biphasic, that is, a mild reaction stimulates tumour growth although a strong one is cytotoxic. It may be further hypothesized that in the early evolution of tumours, when the immune response is incipient and therefore weak, direct lymphocyte and target cell interaction stimulates the tumour cell and intensifies the release of soluble tumour antigen. Acting locally, the soluble tumour antigen " inhibits " efficient lymphocyte cytotoxicity and enhancement of nascent tumour occurs. As the intensity of the immune response builds up and the lymphocytes become capable of a cytotoxic tumour effect, a larger mass of tumour presents itself to the host as a result of the previous stimulation of tumour growth. While a more cytotoxic tumour effect may occur, this again releases soluble cell surface antigen and produces feedback " inhibition " of lymphocyte " killer " function. The tumour mass progressively increases in size and at some critical stage there is a transition from local antigen excess to systemic antigen excess which overwhelms the host's immune response.

The phenomenon of feedback inhibition of soluble tumour antigen on lymphocytic " killer " function may explain why in most instances the tumour develops, metastasizes and finally kills the host instead of encountering resistance and being rejected eventually by an immune reaction of the host.

The technical assistance of Mrs K. Steele is acknowledged with thanks. This research was supported by grants from the Medical Research Council and the Cancer Research Campaign. D. M. P. Thomson was in receipt of a Canadian M.R.C. Fellowship.

### REFERENCES

ALPERS, J. H., STEWARD, M. W. & SOOTHILL, J. F. (1972) Differences in Immune Elimination in Inbred Mice—The Role of Low Affinity Antibody. *Clin. & exp. Immunol.*, **12**, 121.

AVRAMEAS, S. & TERNYNCK, T. (1969) The Cross-linking of Proteins with Glutaraldehyde and its Use for the Preparation of Immunoadsorbents. *Immunochemistry*, **6**, 53.

BALDWIN, R. W. & BARKER, C. R. (1967) Demonstration of Tumour-specific Humoral Antibody against Aminoazo Dye-induced Rat Hepatoma. *Br. J. Cancer*, **21**, 793.

BALDWIN, R. W. & GLAVES, D. (1972) Solubilization of Tumour-specific Antigen from Plasma Membrane of an Animoazo-dye-induced Rat Hepatoma. *Clin. & exp. Immunol.*, **11**, 51.

BALDWIN, R. W., EMBLETON, M. J. & ROBBINS, R. A. (1973*a*) Cellular and Humoral Immunity to Rat Hepatoma Specific Antigens Correlated with Tumour Status. *Int. J. Cancer*, **11**, 1.

BALDWIN, R. W., BOWEN, J. G. & PRICE, M. R. (1973*b*) Detection of Circulating Hepatoma D23 Antigen and Immune Complexes in Tumour Bearer Serum. *Br. J. Cancer*, **28**, 16.

BALDWIN, R. W., EMBLETON, M. J. & PRICE, M. R. (1973) Inhibition of Lymphocyte Cytotoxicity for Human Colon Carcinoma by Treatment with Solubilized Tumour Membrane Fractions. *Int. J. Cancer*, **12**, 84.

BIAS, W. B., SANTOS, G. W., BURKE, P. J., MULLINS, G. M. & HUMPHREY, R. L. (1972) Cytotoxic Antibody in Normal Human Serums Reactive with Tumor Cells from Acute Lymphocytic Leukemia. *Science, N.Y.*, **178**, 304.

BRAWN, R. J. (1971) *In vitro* Desensitization of Sensitized Murine Lymphocytes by a Serum Factor (Soluble Antigen?). *Proc. natn. Acad. Sci. U.S.A.*, **68**, 1634.

CUATRECASAS, P. (1970) Protein Purification by Affinity Chromatography. Derivatizations of Agarose and Polyacrylamide Beads. *J. biol. Chem.*, **245**, 3059.

CURRIE, G. A. (1973) Effect of Active Immunization with Irradiated Tumour Cells on Specific Serum Inhibitors of Cell-mediated Immunity in Patients with Disseminated Cancer. *Br. J. Cancer*, **28**, 25.

CURRIE, G. A. & BASHAM, C. (1972) Serum-mediated Inhibition of the Immunological Reactions of the Patient to his own Tumour: a Possible Role for Circulating Antigen. *Br. J. Cancer*, **26**, 247.

CURRIE, G. A. & Gage, G. O. (1973) Influence of Tumour Growth on the Evolution of Cytotoxic Lymphoid Cells in Rats Bearing a Spontaneously Metastasizing Syngeneic Fibrosarcoma. *Br. J. Cancer*, **28**, 136.

DAVIS, B. F. (1964) Disc Electrophoresis—II Method and Application to Human Serum Proteins. *Ann. N.Y. Acad. Sci.*, **121**, 400.

GREENWOOD, F. C., HUNTER, W. M. & GLOVER, J. S. (1963) The Preparation of $^{131}$I-labelled Human Growth Hormone of High Specific Radioactivity. *Biochem. J.*, **89**, 114.

GUTTERMAN, J. U., MAVLIGHT, G., MCCREDIE, K. B., BODEY, G. P., FREIREICH, E. J. & HERSH, E. M. (1972) Antigen Solubilized from Human Leukemia: Lymphocyte Stimulation. *Science, N.Y.*, **177**, 188.

HELLSTRÖM, K. E. & HELLSTRÖM, I. (1969) Cellular Immunity against Tumour Antigens. *Adv. Cancer Res.*, **12**, 167.

HELLSTRÖM, I., HELLSTRÖM, K. E. & SJÖGREN, H. O. (1970) Serum Mediated Inhibition of Cellular Immunity to Methylcholanthrene-induced Murine Sarcomas. *Cell. Immunol.*, **1**, 18.

HELLSTRÖM, I., SJÖGREN, H. O., WARNER, G. & HELLSTRÖM, K. E. (1971) Blocking of Cell-mediated Tumour Immunity by Sera from Patients with Growing Neoplasms. *Int. J. Cancer*, **7**, 226.

HOLMES, E. C., KAHAN, B. D. & MORTON, D. L. (1970) Soluble Tumor-specific Transplantation Antigens from Methylcholanthrene - induced Guinea Pig Sarcomas. *Cancer, N.Y.*, **25**, 373.

LEWIS, M. G., IKONOPISOV, R. L., NAIRN, R. C., PHILLIPS, T. M., FAIRLEY, G. H., BODENHAM, D. C. & ALEXANDER, P. (1969) Tumour-specific Antibodies in Human Malignant Melanoma and their Relationship to the Extent of the Disease. *Br. med. J.*, iii, 547.

MELTZER, M. S., LEONARD, E. J., RAPP, H. J. & BORSOS, T. (1971) Tumour-specific Antigen Solubilized by Hypertonic Potassium Chloride. *J. natn. Cancer Inst.*, **47**, 703.

NATHENSON, S. G., SCHWARTZ, B. D. & CULLEN, S. E. (1972) *Membranes and Viruses in Immunotherapy*. New York: Academic Press, Inc. p. 117.

OLD, L. J. & BOYSE, E. A. (1964) Immunology of Experimental Tumours. *A. Rev. Med.*, **15**, 167.

PREHN, R. T. (1972) The Immune Reaction as a Stimulator of Tumor Growth. *Science, N.Y.*, **176**, 170.

RUOSLAHTI, E. & SEPPALA, M. (1972) α-Foetoprotein in Normal Human Serum. *Nature, Lond.*, **235**, 161.

SANDERSON, A. R., WELSH, K. I. & CRESSWELL, P. (1971) Involvement of Carbohydrate in the Immunochemical Determinant Area of HL-A Substances. *Nature, New Biol.*, **230**, 8.

SJÖGREN, H. O., HELLSTRÖM, I., BANSAL, S. C. & HELLSTRÖM, K. E. (1971) Suggestive Evidence that the Blocking Antibodies of Tumour-bearing Individuals may be Antigen–Antibody Complexes. *Proc. natn. Acad. Sci. U.S.A.*, **68**, 1372.

SPRINGER, T. A. & STROMINGER, J. L. (1973) Detergent Solubilized HL-A Antigen from Cultured Lymphocytes. *Fedn Proc.*, **32**, 1018 (abstract).

THOMSON, D. M. P., KRUPEY, J., FREEDMAN, S. O. & GOLD, P. (1969) The Radioimmunoassay of Circulating Carcinoembryonic Antigen of the Human Digestive System. *Proc. natn. Acad. Sci. U.S.A.*, **64**, 161.

THOMSON, D. M. P. & ALEXANDER, P. (1973*a*) A Cross Reacting Embryonic Antigen in the Membrane of Rat Sarcoma Cells which is Immunogenic in the Syngeneic Host. *Br. J. Cancer*, **27**, 27.

THOMSON, D. M. P., STEELE, K. & ALEXANDER, P. (1973*b*) The presence of Tumour-specific Membrane Antigen in the Serum of Rats with Chemically-induced Sarcomata. *Br. J. Cancer*, **27**, 35.

THOMSON, D. M. P., ECCLES, S. & ALEXANDER, P. (1973*c*) Antibodies and Soluble Tumour-specific Antigens in Blood and Lymph of Rats with Chemically Induced Sarcomata. *Br. J. Cancer*, **28**, 6.

VAN ROOD, J. J., VAN LEUVEN, A. & VAN SANTEN, M. C. T. (1970) Anti-HL-A2 Inhibitor in Normal Human Serum. *Nature, Lond.*, **226**, 366.

WEBER, K. & OSBORN, M. (1969) The Reliability of Molecular Weight Determinations by Dodecyl Sulfate-polyacrylamide Gel Electrophoresis. *J. biol. Chem.*, **244**, 4406.

WEIR, D. M. & ELSON, C. J. (1969) Antitissue Antibodies and Immunological Tolerance to Self. *Arthr. Rheum.*, **12**, 254.